# Analysis of Graphic Language Expression in Visual Communication Design

**DOI:** 10.1155/2022/2434992

**Published:** 2022-09-09

**Authors:** Haixia Xu, LiLi Shi

**Affiliations:** Qingdao Huanghai College, Qingdao, Shandong 266000, China

## Abstract

With the continuous development of society, only relying on words can no longer satisfy the mutual communication between people and countries. In the background of the data era, graphics language also meets more opportunities and challenges, so it is of great significance to transform data into the graphics language. This paper mainly designs a set of a data-based intelligent system. Through the research of multi-dimensional data visualization, firstly, the high-dimensional data is processed to reduce the dimension, so as to get the lower dimensional data, and then the low-dimensional data is transformed into a more intuitive type of graphic markers, so that users can understand the high-dimensional data more intuitively. The research results show that the system can provide users with the most appropriate label type and save users more time and energy. Data visualization has different experiences for different users, so it is a very important challenge to make users feel that pivoting is as effective as possible.

## 1. Introduction

With the development of social economy, in some specific cases, using graphics to express information is more attractive and persuasive than traditional language. Graphics can more intuitively convey the deeper meaning of words to the audience, and graphics can also serve as the ideological carrier of designers. It is a very important way of communication between people [1]. 'Today's society can be said to be a data-based society. The deeper meaning of numbers needs to be mined. However, it is difficult for us to mine some very useful hidden information directly from the data. At this time, we need to use some commonly used charts to make the data more intuitive in front of people. However, at present, data visualization also faces a variety of question [2]. For example, many data visualization lacks intuitiveness, users still need to draw reports, users do not know what kind of charts they want, so in general, the utilization of face-to-face data visualization is not high [3]. Bertin mentioned the information of visual coding in his book graphic semiotics, and the graphics are divided into graphics content and graphics carrier.

This paper mainly studies a set of an intelligent recommendation algorithm, which only needs the user to provide some field information, and then it can recommend the more appropriate graphics markup for the user. Firstly, this paper improves the merging operator of the graphics language, then improves the modified graphics language, generates the perspective table of the graphics language and finally sets the graphics according to the recommended markup type Plan.

The innovation of this paper is mainly in two aspects. On the one hand, it designs and implements a system of transforming data into graphic language, which is mainly used in the case that users want to know more about some data, and this system can automatically describe the graphics intelligently. On the other hand, when users understand less about the data they want to know, this system can be used. Users recommend tag types, which can speed up the search and utilization of data.

This paper is mainly composed of four parts. The first part is about the background of the graphics language in the era of data. The second part is about the research of the graphics language at home and abroad. The third part is about the theoretical research of the generation method of the graphics language, from which the corresponding recommendation method of the graphics mark is obtained. The fourth part is about the research method of using the graphics language. In practice, the tag type recommendation system of graphic language is obtained.

## 2. Related Work

In China, graphics language comes from the need of transmitting information in our daily life. Ali et al. believed that communication design, as an important tool for sustainable social propaganda, has not been fully explored in both academic and creative practice all over the world [4]. Leeuw et al. believed that there are numerous effective measurement tools in social science research, including positive and negative mixed statements. The purpose of this study is to use dierman's questionnaire survey principle to seek alternative relief for negative discourse [5]. According to Park for those English learners who have been studying in the United States since junior high school or high school, the teaching support that can promote the development of their historical literacy - textbooks and practices - has received little attention. The findings of this study have implications for those practitioners and researchers who are interested in English learners, graphic novels, and history literacy [6]. Pampoulou found that more and more research projects show that graphical symbols with linguistic features are one of the tools that professionals support students in the inclusive school environment. However, few studies have investigated the cooperation between professionals who use these symbols in these situations. Therefore, the purpose of this study is to explore the factors that promote or hinder the cooperation between speech and language therapists and school staff who use graphic symbols [7]. Mooij et al. found that ECoG of high gamma activity induced by language task is considered a more patient-friendly cortical electrical stimulation map (ESM), which is the gold standard of the preoperative language map for epileptics [8]. Arya et al. studied and compared the topography of high gamma modulation (HGM) and conventional electrical stimulation mapping (ESM) in the language localization process before the operation of drug-resistant epilepsy in children. The former did not need the cooperation of patients when listening to the story, and the latter used the image naming task. It is found that if possible, additional language tasks can be added to improve the accuracy of localization diagnosis [9]. Roan thought that Zhao Meizhi's drawing of new color lines: the graphic narration of transnational Asian Americans is a gratifying contribution to the graphic narration of Asian Americans and the academic research of comic writers. As Zhao pointed out in her introduction, this comic book is not intended to “fill in” gaps in the field of comic research [10]. Schlosser et al. used the graphic symbol set of an autistic language program, an independent *t*-test showed that dynamic symbols are easier to identify than static symbols, encouraging clinicians to use animation when introducing graphic symbols representing verbs [11]. Alhanbali et al. found that the response of the magnetic signal and electroencephalogram (M/EEG) signal to voice degradation has been confirmed by several recent studies. In the current EEG study, the acoustic characteristics of stimuli have been changed, and the test is classified according to the correctness of the listener's oral response [12]. Riskedahlpointed out through the study of language strategy that these text products contain the remains of controversial populism, national status, and belonging ideology. When these ideologies are placed in specific geographical symbol areas in the urban political landscape, they gain special significance [13]. Fakharkonandeh found that multimedia and poetic styles are not only vividly reflected in Buck's language but also in his highly complex audio-visual production aesthetics. This article explores language/text, image/photography, and perspective in aesthetics and ethics [14]. Chholak et al. evaluated the language laterality of candidates for epilepsy surgery before operation, the current “gold standard” is the “amobarbital procedure” (IAP) in the carotid artery. This simple magnetoencephalogram paradigm shows the feasibility and is non-invasive in identifying the left hemisphere language advantage of candidates for epilepsy surgery [15].

The research is divided into five parts. The first part analyzes the development of data visualization and the various problems it still faces. The second part analyzes the research on graphic language at home and abroad. Through the study of graphic language, we can know that graphics are formed in life and are the result of people's imagination. The third part is the theoretical research of the graphics language generation method, from which the corresponding graphics marking recommendation method is obtained. The fourth part is about the research method of using graphics language in practice and obtains the tag recommendation system of the graphics language. Finally, the full text is summarized. The research results show that the system is very convenient for customers and allows customers to find the appropriate type of tag display data in a short time. The system can provide users with the most appropriate label type and save users more time and energy.

## 3. Research Methods of the Graphic Language in Visual Communication

### 3.1. The Development of the Graphic Visual Language

Graphics is a very important form of expression in visual communication design. It is a way of human communication and emotional expression. The characteristics of graphics itself make it occupy a very important position in the development of society. It has more visual characteristics than language expression. It can cross the cultural and language communication difficulties in different places. Graphics with its own personal art and its own charm infect designers and the public and carry out effective information communication. Graphic symbols use a variety of symbols to represent different graphics. Bertin mentioned the information of visual coding in the book of graphic semiotics and divided the graphics into the content of graphics and the carrier of graphics. The content of graphics is mainly used to express the information that the graphics want to transmit to the public, while the carrier of graphics is mainly used to express the specific symbols of graphics.

In our life, we often use a variety of graphic symbols, such as points, lines, faces, and bodies, which are so-called mathematical graphic symbols, mainly used to express various information. Position and retina variables constitute visual variables in visual communication, and graphic symbols can use visual variables to express more information. According to these terms, position variables refer to a certain position of a figure in the three-dimensional space, and the appearance of the figure, for example, the color, size, and position of the figure are retinal variables. It comes from a paragraph of text information in a database. It mainly uses graphic design as a bridge to connect data and the image structure. The collection of all image languages constitutes the graphics language, and any graphic can be expressed by one statement. Generally speaking, the single graphic statement constitutes the inter graph, and the complex graphic statement constitutes the complex graph. The author also points out that two essential points in the performance of a graphic language are expressiveness and effectiveness. What can be expressed is that a graph can be expressed with data, and what is effective is that the graph can completely express the hidden information of data. Therefore, on this basis, the author further designs a set of basic graphic statements and three combination operators, which are biaxial combination, single extraction combination, and mark combination. Double extraction merging refers to the integration of graphs with the same horizontal and vertical coordinates. As the name implies, single extraction merging means to align the images with the same horizontal and vertical coordinates and mark merging means to align the marks in the images.

### 3.2. Visualization of High Dimensional Data in the Graphic Visual Language

When dealing with various kinds of data, we often use various models to deal with data. In our life, we can easily understand the two-dimensional and three-dimensional structure of space, but it is difficult to understand the higher dimensional data. Therefore, in order to make people better understand the higher dimensional data, we usually base it on the lower dimensional data and then bind the higher dimensional data with color, size, appearance style, etc. There is also a problem with this way of processing, that is, when the data dimension is particularly high, it will reduce the readability of the data that can be seen intuitively. Therefore, we need to use other research methods to process the high-dimensional data, so that the dimension can be reduced and the low-dimensional space can be read intuitively. Next, we mainly recommend two methods to make high-dimensional data and low-dimensional data. They are the scatter matrix, which mainly recommends the graphic design in the text in the form of inspiration, while table perspective is mainly used to reduce high-dimensional data to low-dimensional data.

Scatter diagram projects high-dimensional data into the coordinate axis of low-dimensional space, so that the distribution of data can be observed intuitively, and the relationship between data can be understood, for example, there are *m* variables. Using the scatter diagram matrix, the relationship between two variables can be observed intuitively. Table is a common method used to display multi-dimensional data. A column and a row in the table represent a data attribute and a data record, respectively. Therefore, none of the cells in the table represents the specific value of the row data belonging to the column data, as shown in Table 1 below.

The expression form of the table lens is similar to that of the table. The difference is that the value in the table lens cannot be the data, and the data graph can be used to represent the size of the data. Therefore, the data bar can be read intuitively to understand the relative size of the data. This method is clearer and easier to understand than the table, as shown in Table 2.

The rows and columns in each table represent the attributes of data records and data, respectively, so the table is only suitable for simple data presentation, but we often process the data and make statistics to find the hidden information of the data, so the tables between them cannot meet our needs. At this time, we will process the data table. For example, we can store some of the data in the table in rows and columns, and some of the data can be applied by ourselves, so we can make a perspective table, as shown in Table 3. In a pivot table, every content in the table represents a value with different meanings. Using the table lens, we can use data bars or dots of other shapes to represent this value. We can also put other graphs in the table in the pivot table, such as bar graph, column graph, and pie chart, which can make us more profound and can understand the implications of this data.

Table 3 shows the types of customers and the total number of enterprises and customers in Northeast and Southwest China. According to the comparison, the customer categories and the total number of enterprises in the Northeast are larger than those in the southwest.

### 3.3. Research Flow of the Intelligent Data System

**Figure 1:**
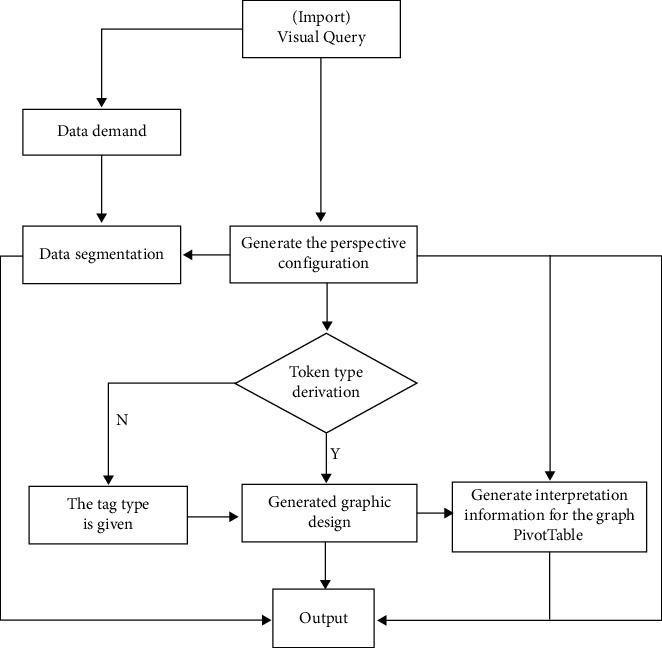
The graph pivot table configures the generated workflow.

This paper deals with the generation of a graphic pivot table and the recommendation of a single field and multi-field configuration. The former is mainly to generate a certain visual query pivot table, while the latter is mainly to determine a field and then put it into the pivot table, so as to form a new pivot table. In this way, the visual expression effect of the query perspective table is more intuitive and clear. As shown in Figure 1 below, Figure 1 means to input visual query into the system, and corresponding configuration information required by a pivot table will be output, as well as the working direction of the pivot table. Each arrow in the figure means the direction of each data.

The first step of this data flow is to input a visual query and then use the information to search in the database to get the structure configuration needed to form the pivot table and then use the configuration to cut the data table in, so as to form a small cell, at the same time, generate their own graphics, so as to further generate the pivot table, and finally get the structural configuration of the table set and so on as well as data table explanation information.

**Figure 2:**
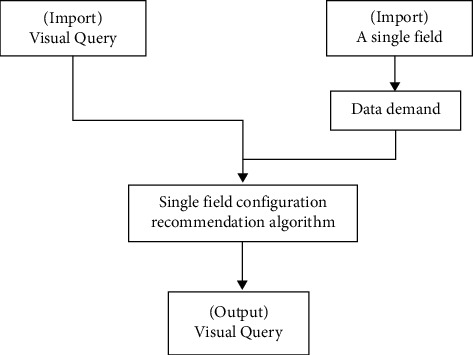
Multiple fields configure the recommended workflow.

If the user wants a new field, he can input a visual query at the same time. After input, the system will process the data and execute the single field calculation process according to the user's requirements. The similar multi-field process needs two steps. First, according to the input field information, the system will search for the appropriate chart to display the information. Second, according to the figure obtained in the first step, a suitable chart to display the configuration information of the pivot table is specified. The specific process is shown in Figure 2.

The main purpose of this process is that when users input multiple field information, the system will find some graphs and tables suitable for displaying these information during data search and then further optimize these graphs and sort the optimized results. Therefore, when users want a certain kind of graphs and tables, first use the sorted data to observe whether these charts are suitable for display and utilization, if appropriate, the next step of the optimal recommended configuration algorithm will be carried out, and if not, the search will continue, and the final result will find a suitable chart to represent the input information.

## 4. Research on the Recommendation System of the Perspective Table of the Graphic Language Based on Visual Communication

The rest of a pivot table has been determined. In the absence of a certain tag, selecting a tag type with a large visual difference will give users a more direct visual effect. All of us need to help customers to draw up the final tag type, so we first understand the tag type in some scenarios and then recommend it according to the information provided by users and various tag types. In general, the commonly used marker types are point, line, face, text, bar, pie chart, Gantt chart, and so on. These types of tags are often used in different scenarios. Column chart is widely used in drawing. It is mainly used to compare columns with a certain height with others or to represent continuous trend change. Lines connect several points, which are also lines in our life. So lines are mainly used to represent the change of a variable over time. Area is the area formed on the coordinate axis and by lines and coordinate axes. Area is mainly the area of some areas compared by people in daily life. Points are used to express the connection between two dependent variables. Words are mainly used to express the meaning of the text itself and not much. They are mainly used in some tables. Sector chart is a bar chart in the coordinate system, and pie chart is a surface chart. Display the cutting of fan-shaped area, and visually display the proportion of the part. There are not many ways to deal with data in the Gan column chart, which is mainly used to indicate that some items are dealing with the penalty ball and the end point together. Filling map is mainly used when *XY* axis is the figure drawn by longitude and latitude, respectively.

In some cases, when the *XY* axis has no data field information, we can only recommend different tags according to the intuitive vision. Therefore, in this case, pie chart is the most appropriate choice. It can provide users with more information prompts, but not all cases use a pie chart. If there is field prompt information of color attribute but no size and angle prompt, then we can only get a pie chart with the same area. When we encounter this situation, we need to use text tags. Moreover, when the size and angle use discrete fields and we cannot use them, it is more appropriate to use the text as the label type than a pie chart. From the above discussion, we can get the mark type we should use when there is no specific meaning in the *XY* axis, as shown in Table 4 below, where *y Es* indicates that there is a field information that encodes the user's visual attributes. No indicates the opposite of yes. Any indicates whether there is any field information. When there is a specific demand, it should be noted.

When there is only one existing field on *XY* axis, we call it a single axis. Then the mark type is determined by visual attribute and single axis end field together. At this time, the best mark type recommended by the system to users is sector. When the shape attribute is a discrete field, the most suitable type of tag is scattered, while other types of tag cannot be used to represent the shape and angle. Bar chart is most suitable for representing a continuous field information, and the initial point of the Gantt bar is determined by a continuous field because it can be cut, and it can be used to replace the bar as the most suitable marker type. Finally, there will be a situation where the end field of a single axis is scattered and suitable, and there is no other intuitive information in the outside world. At this time, we can use text as the marker type, and if there is other intuitive information, then we are willing to use other marker types better than that. For example, when the field information is color, we can use the sector chart to represent and can to represent visual information by the color or size of a bar chart. From the above analysis, we get the recommendation of type mark when it is in a single axis, where yes represents the visual information, no represents no prompt information, any represents whether there is prompt information, and if there is any other information, please note it.  Table 5 is the recommended table of single-axis label types.

The data is reduced from high-dimensional space to low-dimensional space, and similar features will be merged because of the variance. Therefore, the data will be reduced and the number of features will be reduced, which is conducive to preventing the occurrence of overfitting phenomenon. But PCA is not a good method to prevent overfitting. When preventing overfitting, it is best to regularize the data. In the last case, when there is prompt information on the *XY* axis, it is called the double axis. The last prompt information on the *X* axis and *Y* axis determines the final marker type. Similar to the single axis above, when there are continuous fields representing prompt information, pie is the recommended marker for comparison at this time. If it is discrete information, it is more suitable for scatter. If the last prompt information in both directions of the *XY* axis is continuous or discrete, it is more suitable for scattering at this time. When one of the last prompt information of *XY* axis direction is a continuous measurement and the other is a continuous dimension, we are more suitable to express it with a line. When both of the *XY* axis are discrete prompt information, it is more appropriate to use text to express at this time, but if there is prompt information such as color, the pie chart is more appropriate. In addition, we can also use bar to express. It is also important to note that if the last hint of the *XY* axis is geolongitude or dimension, then the most appropriate marker type is the filled map. Therefore, from the above analysis, we can get a recommended table in Table 6 below. Yes means there is visual information, no means there is no prompt information, any means there is no prompt information, if there is any other information, you can comment.

## 5. Conclusion

This paper designs a set of the automatic recommended graphics type annotation system, which mainly processes data and uses the field information provided by users to search big data in the system. Then recommend the most appropriate graphic mark to the user, so that the data can be displayed in the form of graphics in front of the user. In this way, users can more intuitively observe the laws behind the data, so as to deepen our understanding of the logarithm. The research results show that this system is very convenient for customers and allows customers to find the appropriate type of mark display data in a short time. The system can provide users with the most appropriate label type, saving users more time and energy. Data visualization has different experiences for different users, so it is a very important challenge to make users feel that pivoting is as effective as possible. However, the system also has some shortcomings, such as no more in-depth analysis of the data. It only recommends tag types to users but does not dig deeper into the meaning of data hiding, which requires further exploration by humans and is worthy of our follow-up research.

## Figures and Tables

**Table 1:** Table diagram.

Nation	Area	Customer category	Product category	Sale	Profit
China	Northeast	Enterprise	Work	32768	3567
China	Northeast	Enterprise	Technology	46902	4873
China	Northeast	Customer	Work	68925	5326
China	Northeast	Customer	Technology	92743	8145
China	Southwest	Enterprise	Work	10256	1476
China	Southwest	Enterprise	Technology	25748	2274
China	Southwest	Customer	Work	22973	2898
China	Southwest	Customer	Technology	54788	6073

**Table 2:** Schematic diagram of table perspective mirror.

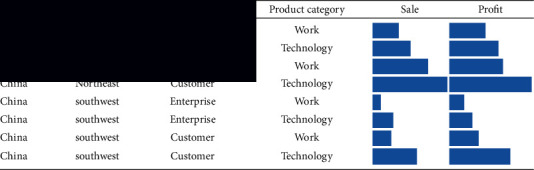

**Table 3:** A data perspective represents intent.

Nation	Area	Customer category	
		Enterprise	Customer
China	Northeast	32768	46902
		68925	92743
	Southwest	10256	25748
		22973	54788

**Table 4:** No axis tag type recommendation table.

Color	Size	Angle	Shape	Lable	Tag type
Any	Any	Any	Disperse	Any	Scatter
Yes	No	No	No	Yes	Text
No	Yes	No	No	Yes	Text
No	No	Yes	No	Yes	Text
Any	Disperse	Any	No	Yes	Text
Any	Any	Disperse	No	Yes	Text
Other situations					Pie

**Table 5:** Recommended table for single-axis tag types.

Axis field	Color	Size	Angle	Shape	Lable	Tag type
Yes	Any	Any	Continuous	Any	Any	Pie
Yes	Any	Any	Any	Disperse	Any	Scattet
Continuous measurement	Any	Any	No	No	Any	Bar
Continuous dimension	Any	Any	No	No	Any	GanttBar
Disperse	No	No	No	No	Any	Text
	Dimensionality	Continuous	Any	No	Any	Pie
	Other situations					Bar

**Table 6:** Biaxial tag type recommendation table.

Axis field 1	Axis field 2	Color	Size	Angle	Shape	Lable	Tag type
Yes	Yes	Any	Any	Yes	Any	Any	Pie
Yes	Yes	Any	Any	Any	Yes	Any	Scattet
Continuous measurement	Continuous measurement	Any	Any	No	Any	Any	Scattet
Continuous dimension	Continuous dimension	Any	Any	No	Any	Any	Scattet
Continuous measurement	Continuous dimension	Any	Any	No	No	Any	Line
Time	Continuous measurement	Any	Any	No	No	Any	Line

Disperse	Disperse	No	No	No	No	Any	Text
		Measurement	Any	No	No	Any	Text
		Dimensionality	Continuous	Any	No	Any	Pie
		Other situations					Bar

Disperse	Continuous measurement	Any	Any	No	No	Any	Bar
	Continuous dimension	Any	Any	No	No	Any	GanttBar

Geographic longitude	Geographic latitude	Any	Any	No	No	Any	FilledMap

## Data Availability

The data used to support the findings of this study are available from the corresponding author upon request.
